# Divergent water requirements partition exposure risk to parasites in wild equids

**DOI:** 10.1002/ece3.8693

**Published:** 2022-03-14

**Authors:** Kaia J. Tombak, Laurel A. Easterling, Lindsay Martinez, Monica S. Seng, Liana F. Wait, Daniel I. Rubenstein

**Affiliations:** ^1^ Department of Anthropology Hunter College of the City University of New York New York New York USA; ^2^ 6740 Department of Ecology and Evolutionary Biology Princeton University Princeton New Jersey USA; ^3^ School of Veterinary Medicine University of Pennsylvania Philadelphia Pennsylvania USA; ^4^ The Wildlife Society Bethesda Maryland USA; ^5^ Nomad Health New York New York USA

**Keywords:** equid parasitology, fecal egg counts, gastrointestinal nematodes, helminth transmission risk, Parasite exposure risk, zebra habitat use

## Abstract

For grazing herbivores, dung density in feeding areas is an important determinant of exposure risk to fecal‐orally transmitted parasites. When host species share the same parasite species, a nonrandom distribution of their cumulative dung density and/or nonrandom ranging and feeding behavior may skew exposure risk and the relative selection pressure parasites impose on each host. The arid‐adapted Grevy's zebra (*Equus grevyi*) can range more widely than the water‐dependent plains zebra (*Equus quagga*), with which it shares the same species of gastrointestinal nematodes. We studied how the spatial distribution of zebra dung relates to ranging and feeding behavior to assess parasite exposure risk in Grevy's and plains zebras at a site inhabited by both zebra species. We found that zebra dung density declined with distance from water, Grevy's zebra home ranges (excluding those of territorial males) were farther from water than those of plains zebras, and plains zebra grazing areas had higher dung density than random points while Grevy's zebra grazing areas did not, suggesting a greater exposure risk in plains zebras associated with their water dependence. Fecal egg counts increased with home range proximity to water for both species, but the response was stronger in plains zebras, indicating that this host species may be particularly vulnerable to the elevated exposure risk close to water. We further ran experiments on microclimatic effects on dung infectivity and showed that fewer nematode eggs embryonated in dung in the sun than in the shade. However, only 5% of the zebra dung on the landscape was in shade, indicating that the microclimatic effects of shade on the density of infective larvae is not a major influence on exposure risk dynamics. Ranging constraints based on water requirements appear to be key mediators of nematode parasite exposure in free‐ranging equids.

## Graphical abstract

Exposure risk to fecal‐orally transmitted parasites depends on the spatial distribution of feces and of feeding behavior and can be compounded when multiple sympatric host species share the same parasites. We present evidence that in equid hosts, water dependence is a key determinant of exposure risk to gastrointestinal nematodes because dung density increases with proximity to water access points. Our results indicate that the water‐dependent plains zebra has higher exposure risk than the arid‐adapted Grevy's zebra to shared parasites on a common landscape, skewing the burden of parasite pressure between these hosts with implications for coexistence and habitat suitability.

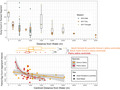

## 1 INTRODUCTION

Host density is a central determinant of the rate of parasite transmission in classic epidemiological models (reviewed in Dobson, 1990). For fecal‐orally transmitted parasites, high host density raises transmission rates by intensifying the common use of the same habitat. Many models assume a straightforward relationship between host density and transmission probability, implicitly or explicitly assuming random defecation and feeding behavior within home ranges (Grenfell et al., 1987). However, both host defecation and feeding behavior can be decidedly nonrandom, altering exposure risk. For example, territorial hosts often use latrines, concentrating their dung in small areas, typically at the territorial periphery (Brashares & Arcese, 1999; Klingel, 1972), whereas most feeding is likely to occur well within territory boundaries. Exposure risk for fecal‐orally transmitted parasites would therefore more accurately be approximated by the density of infective feces in feeding areas than by host density.

Host species that are closely related are the likeliest to harbor the same parasite species (Poulin, 2010). For relatively generalist parasites like helminths, several host species may transmit to one another, raising both infection prevalence and intensity as more host species share the same habitat (Ezenwa, 2003). In such cases, their cumulative dung density becomes the relevant factor for transmission, and interspecific differences in defecation or feeding behavior may then put some species at higher exposure risk than others (Ezenwa, 2004a, 2004b; VanderWaal et al., 2014). This principle may also apply to different classes of conspecifics, distinguished by factors such as age, sex, reproductive status, body condition, or other factors that affect where individuals range and feed. In the present paper, we explore whether intra‐ and interspecific spatial niche partitioning can skew exposure risk to fecal‐orally transmitted parasites among hosts.

As large‐bodied, wide‐ranging, bulk‐feeding grazers (all factors that can increase parasitism; Arneberg et al., 1998), equids host fecal‐orally transmitted gastrointestinal nematodes (GINs) of a striking diversity compared to other ungulates (Bowman, 2003) and compared to other mammals of similar body size (Morand & Poulin, 1998). Two closely related zebra species—the plains zebra (*Equus quagga*) and the Grevy's zebra (*Equus grevyi*)—overlap in range in central Kenya and carry the same set of GIN species but share few with other sympatric ungulates (Titcomb et al., 2020; Tombak et al., 2021). The vast majority of the GIN species equids carry are strongyle nematodes, which have a distinct egg morphology (Foreyt, 2001) but their eggs cannot be identified to genus or species level without the use of genetic analyses. Plains zebras have been found to exhibit higher fecal egg counts (FECs) than Grevy's zebras (Rubenstein, 2010). While FECs give only a rough estimate of relative adult worm burdens in equids (Krecek et al., 1987; Matthee et al., 2000; Nielsen et al., 2010; Scialdo et al., 1982; Scialdo‐Krecek et al., 1983; Wambwa et al., 2004), this interspecific difference may indicate that plains zebras experience higher exposure to GINs.

Plains zebras generally need to drink every day (Fischhoff et al., 2007; Rubenstein, 2010), while Grevy's zebras are more arid‐adapted and drink only every three to five days except during early lactation (Becker & Ginsberg, 1990; Ginsberg, 1989). Grevy's zebras should thus be able to range much farther from water holes and to graze in areas plains zebras and other more water‐dependent ungulates cannot reach. Because water holes serve as attraction points, we expect that dung would be more concentrated in proximity to water (Titcomb et al., 2021) and that plains zebras would be constrained to feed in areas with higher dung density. Active fecal avoidance (choosing to feed on grass swards uncontaminated with dung) is unlikely to be of great influence on exposure risk in zebras. Fecal avoidance is not typical of horses, zebras, or other equids in large pastures or under free‐ranging conditions (Lamoot et al., 2004; Silveira, 2019), and horses readily choose feces‐contaminated swards if they offer even slightly more food than uncontaminated swards (Fleurance et al., 2005). However, the density of infective larvae produced in dung may be spatially variable with potential to skew exposure risk between host species with different ranging patterns. Ambient temperature, rainfall, and evaporation rates are used to predict rates of survival and development of free‐living nematode stages (e.g., the GLOWORM‐FL model; Rose et al., 2015), but microclimatic conditions, such as sun exposure, may override these conditions measured at a larger scale to produce spatial heterogeneity in exposure risk. Experiments have shown equine strongyles to be generally heat‐resistant and desiccation‐susceptible until the third (infective) larval stage, when they develop a hard cuticle and become desiccation‐resistant and heat‐susceptible. However, heat‐sensitivity was tested only for temperatures up to 38°C and data are lacking on desiccation susceptibility for strongyle eggs (Nielsen et al., 2007). Here, we expand on these experiments to understand microclimatic effects on strongyle egg development in a semi‐arid, tropical habitat.

The objectives of the present paper are threefold: (1) to investigate the potential skew in exposure risk that ranging behavior imposes on hosts at the interspecific level, and (2) at the intraspecific level, and (3) to test whether microclimatic effects on infective larval density may further skew exposure risk. We determined how dung density was distributed with respect to water and related this to ranging and feeding patterns in the arid‐adapted Grevy's zebra and the mesic‐adapted plains zebra. We then assessed how the proximity of home ranges to water compared between the species and between sexes, and how this proximity affected nematode egg counts in feces. We also studied the susceptibility of nematode eggs to heat and desiccation and their likelihood of developing into infective larvae through field experiments and determined rates of feeding in sun and in shade for the two zebra species.

## 2 MATERIALS AND METHODS

### 2.1 Study site

We conducted our study at Mpala Ranch, Laikipia District, Kenya (190 km^2^, 00°17′N, 36°53′E, elevation = 1700–2000 m), a private ranch and conservancy that spans a transition from black cotton soil with high clay content in the southwest to red sandy loam soil to the northeast in a semi‐arid savanna ecosystem (Young et al., 1997). We focused this study on the southern portion of Mpala, an area of about 100 km^2^ (Figure S1). The rains are roughly bimodally distributed over the year with peaks around April–May and October–November and, in some years, an additional peak around July. The average annual rainfall for the past decade is 630 mm (Mpala Research Centre; Caylor et al., 2018).

### 2.2 Interspecific spatial niche partitioning and bias in exposure risk

**FIGURE 1:**
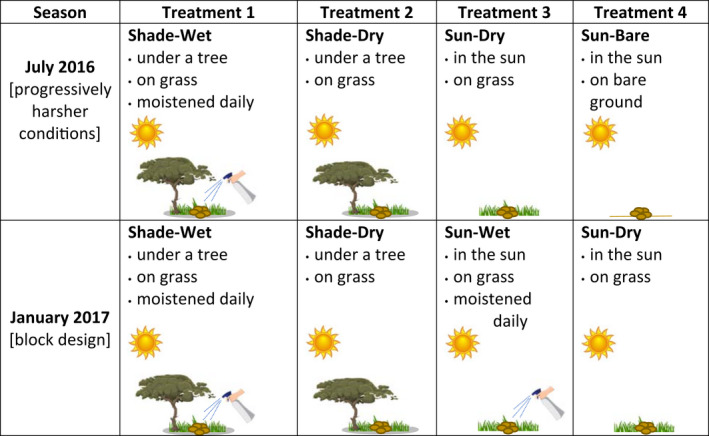
Experimental design for each season of egg embryonation field experiments

To determine the relationship between dung density and distance from water, we conducted long‐distance dung transects, walking away from water points, counting all zebra dung piles along 250 m × 6 m transect segments that ran at increasing intervals of 200‐1000 m for up to a total transect length of 4 km. The interval between transect segments and the total transect length varied by season (see Figure 1, top panel). To capture seasonal variation, 15 transects were run in the July 2016 wet season, six transects in the January 2017 dry season, and three transects, run in duplicate a few days apart to verify repeatability, during the March 2017 drought. Dung piles usually occur singly, but occasionally zebras defecate on top of the dung of another zebra ("over‐marking"; Moehlman, 1985); whenever we encountered a dung pile roughly twice as large as the typical dung pile, it was counted as two dung piles. No large dung piles, used by Grevy's zebra territorial males regularly to demarcate their territory (Klingel, 1972), were encountered in our transects. A negative binomial model was fit with the total zebra dung count in each transect segment (excluding those from the second run of duplicate transects) as the outcome variable and distance from water as the predictor, with data pooled across seasons, using the *glmmTMB* package and model fit was assessed using the *DHARMa* package (Brooks et al., 2017; Hartig, 2018).

To compare dung density exactly where plains and Grevy's zebras grazed, we also counted dung piles in short‐distance transects of 25 m × 2 m, run in March 2017 and June–July 2017 within two days after grazing was observed during routine zebra censuses on Mpala. We used a Kruskal–Wallis test and a post hoc Dunn's test to compare dung density between Grevy's grazing sites (*n* = 10), plains grazing sites (*n* = 31), and random points generated with QGIS on a map of Mpala (*n* = 27).

To map out home ranges, we conducted targeted zebra censuses by driving systematic loops around Mpala Ranch in two field seasons—June–July 2016 and June–July 2018—both of which fell in the early dry season although there was unusually high rainfall in June 2018. Whenever zebras were seen, we photographed each individual and recorded the species, group size and composition, GPS location, and activity of the majority of group members (namely, whether or not they were grazing). Census photographs were processed through a stripe‐recognition program (Crall et al., 2013; Parham et al., 2018) so that individuals in each sighting could be identified. For any zebras seen on at least five different days within a field season, home ranges were constructed using minimum convex polygons (MCPs) and the distances between each MCP centroid and the closest water access point were calculated using QGIS software (version 3.4.7—Madeira; QGIS Development Team, 2018). We compared these distances for Grevy's and plains zebras using a Wilcoxon rank sum test. Here, we refer to the area of habitat used by an animal as its home range, which for breeding males in Grevy's zebras is likely to consist mostly of their territories, although they are known to make frequent excursions outside of the territories they defend (Ginsberg, 1988).

### 2.3 Intraspecific spatial niche partitioning and bias in exposure risk

We related ranging behavior to parasitism among individuals of each species. During zebra home range censuses in the 2016 and 2018 field seasons, we collected fresh dung samples opportunistically from all individuals from which we obtained home range data to measure FECs. We used a modified McMaster salt flotation technique with a 3 g subsample taken from fecal samples homogenized in a plastic bag, and with a minimum detection level of 50 eggs/gram (Ezenwa, 2003; Herd, 1992). Fecal egg counts are crudely related to adult worm burden in equids (Nielsen et al., 2010), and we expected FECs to be negatively related to the distance of a zebra's home range centroid from water. After plotting the data, we tested simple linear and nonlinear (negative exponential) models with the centroid distance from water as the predictor variable and the mean FEC for that individual during the field season as the outcome variable and reported results from the better‐fitting model, as assessed with Q‐Q plots, for each species. To incorporate a test of an interspecific difference in FEC, we additionally ran a linear model on the full dataset with FEC as the outcome variable and centroid distance from water, species, and their interaction as the predictor variables.

### 2.4 Microclimatic effects on bias in exposure risk

#### 2.4.1 Frequency of shade in feeding and defecation sites

We wished to determine whether Grevy's and plains zebras differed in how often they grazed in the shade (i.e., under a tree), because we expected dung in the shade to be more infectious and interspecific differences in exposure risk may arise from the microclimate at feeding sites. In January 2017, 61 scan samples with activity noted every 3 min over a 30‐min session were obtained on individual zebras of both species encountered during censuses (Altmann, 1973). When grazing, we noted whether the animal was in the shade or the sun. We ran Wilcoxon rank‐sum tests to compare the two species in the proportion of grazing time spent in the sun. To determine the proportion of zebra dung piles in the shade vs. the sun, we used data from our long‐distance transects run in January 2017, when it was recorded whether each dung pile was found in sun or shade (see Section 1; counts from the second replicate were used because slightly more dung was spotted in the second replicate for two of the three transects).

#### 2.4.2 Embryonation experiments

Lastly, we expected dung in the shade of a tree to be more infective than dung in the sun because of the milder temperature and lower evaporation rates in shade, promoting nematode egg survival and development (Nielsen et al., 2007). We collected the entire pile of fresh feces from several zebras of both species and for each sample, we thoroughly massaged the dung in a bag to homogenize the eggs within it. We extracted a subsample of 3 g to conduct initial egg counts via the modified McMaster technique. We rolled the rest of the sample into five balls of the same size as zebra fecal pellets (or horse pellets). We arranged these in a tight ring in the field, with each ring composed of balls of the same sample and subjected to one of four treatments. Two sets of experiments were run, one in July 2016 (the wet season), testing a series of progressively harsher conditions (under a tree, on grass, and moistened daily with three spritzes from a water bottle; under a tree, on grass; in the sun, on grass; in the sun, on bare ground), and another in January 2017 (the dry season) using a block design to test the effects of sun/shade and moisture (under a tree, on grass, and moistened daily as above; under a tree, on grass; in the sun, on grass, and moistened daily; in the sun, on grass; Figure 1). The experiments in the wet season were run in two back‐to‐back sessions for six days each and with five replicates per treatment in each session, while the dry season experiments were run in one intensive session over five days, with ten replicates per treatment. In the dry season experiments, we were able to take multiple FEC measures for each sample, producing a minimum detection level of 17 eggs/g, and the average FEC was used for each sample.

Every day, we removed a ball from each ring for egg counts, generating separate FECs for eggs that were still unembryonated (without a visible worm inside) and eggs that were embryonated (with a small unhatched worm visible inside). Each day, we calculated the number of embryonated or unembryonated eggs relative to the initial egg count for each sample and computed the area under the curve as the number of unembryonated eggs fell and the number of embryonated eggs rose and subsequently fell over time using the *AUC* function in the *DescTools* R package (Signorell et mult. al., 2019). We measured the surface temperature of dung at several time points in the day for each treatment using a laser gun in the dry experimental season. We ran a generalized least squares (GLS) model to determine the effect of moistening, shade, and hour of the day, as well as the interaction between the latter two, on surface temperatures with a weighted identity function. Temperature and relative humidity at Mpala Research Centre were compared for the two experimental field seasons using Wilcoxon rank sum tests (Caylor et al., 2017).

R Studio version 1.0.143 was used for all statistical analyses (R Core Team, 2018), and we set alpha to 0.05 for determining significance.

## 3 RESULTS

### 3.1 Interspecific spatial niche partitioning and bias in exposure risk

**FIGURE 2:**
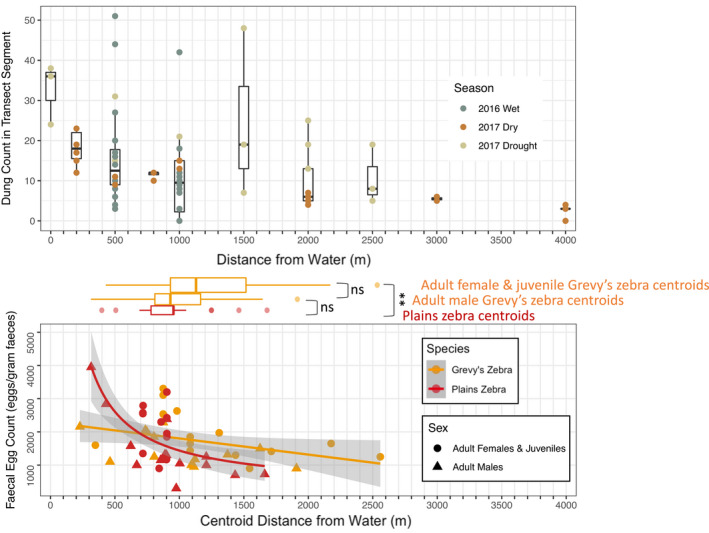
Dung density decreases with distance from water (top panel) as do FECs for both Grevy's and plains zebras the farther their home range centroids lie from water (bottom panel). Horizontal boxplots represent the distribution of home range centroids from water

Our long‐distance dung transects revealed that dung density decreased nonlinearly with distance from water (negative binomial model: *p* < .01, est = −0.036, Z = −2.78, *N* = 62 transect segments; Figure 2). Counts from corresponding segments of duplicated transects were well correlated, suggesting high repeatability in the long‐distance transects (*p* < .001, *F* = 60.28, adj.*R* ^2^ = .82). In total, we detected 887 dung piles on these long‐distance transects (or 1015, counting replicate transects).

In 2016, we mapped the home ranges of 16 Grevy's zebras, all of which were in unstable groups (mean of 18.1 sightings per individual, SE = ±2.5) and 20 plains zebras in 18 stable groups (mean = 11.4 ± 1.4 sightings). In 2018, we mapped those of 12 Grevy's zebras in six stable groups (12.3 ± 2.7 sightings) and five plains zebras in one stable group (10 sightings). Grevy's and plains zebras overlapped in range on transitional soil near the middle of Mpala but their home ranges extended out largely in different directions, Grevy's zebras to the red soil in the east and plains zebras to the black cotton soil in the southwest (Figure S1).

Home range centroids were marginally farther from water points for Grevy's zebras than for plains zebras (W = 454, *p* = .065, *N*_GZ_ = 28, *N*_PZ_ = 25). However, unlike breeding plains zebra males, which form stable groups with females, Grevy's zebra males compete to hold territories close to water because it attracts females (Rubenstein, 1986). Accordingly, when Grevy's zebra males were excluded, we found that the centroids of Grevy's zebras were significantly farther from water than plains zebras (W = 134, *p* < .01, *N*_GZ females + juveniles_ = 15, *N*_PZ_ = 25; Figure 2). Dung counts from short‐distance transects within plains zebra grazing areas were significantly higher than at random points in the landscape, while dung counts in Grevy's zebra grazing areas did not differ from background levels. Grevy's zebra grazing sites had marginally fewer dung piles than plains zebra grazing sites (Kruskal–Wallis χ^2^ = 20.9, *p* < .001, partial statistics from Dunn's *post hoc* test: Grevy's‐Plains Z = −2.08 *adj*.*p* = .074, Grevy's‐Random Z = 1.18, *adj*.*p* = .24, Plains‐Random Z = 4.53, *adj*.*p* < .0001, *N*_GZ_ = 10, *N*_PZ_ = 31, *N*_Random_ = 27). A total of 421 dung piles were counted across all short‐distance transects.

### 3.2 Intraspecific spatial niche partitioning and bias in exposure risk

We collected 297 fecal samples from 36 zebras in the 2016 field season and 112 samples from 17 zebras in the 2018 field season (mean = 7.7 samples/zebra, range = 2 to 19 samples). All fecal samples contained strongyle eggs (prevalence was 100%). The relationship between fecal egg counts and the distance from home range centroids to water was negative for both species. While a linear regression fit the data for plains zebras, the pattern was better described by an exponential decay model (*p* < .001, *F* = 18.45, adj.*R* ^2^ = .42, *N* = 25). For Grevy's zebras, FEC was best predicted by a linear model (*p* < .05, slope = −0.49, *F* = 4.93, adj.*R* ^2^ = .12, *N* = 28; Figure 2). Overall, FECs were similar in plains zebras (mean = 1744 eggs per gram or “epg,” SD = 910) and Grevy's zebras (mean = 1738 epg, SD = 654). However, when the two species were compared using a linear model, species had the greatest effect on FECs (plains zebras had higher FECs than Grevy's zebras; slope = 1244.5, *p* < .05) and the species x centroid distance interaction was also significant (plains zebras had a steeper response than Grevy's zebras; slope = −1.53, *p* < .01), while the centroid distance effect on FECs became marginally significant (slope = −0.49, *p* = .053; model adj.*R* ^2^ = .26, *p* < .001, *F* = 7.06; likely due to highly variable FECs close to water in the pooled data; Figure 2). A plot of the best‐fit models for each species clarified these patterns, revealing that when home ranges are close to water, plains zebras have higher FECs than Grevy's zebras, but that as FECs for both species decrease with greater distance from water, those of plains zebras decrease more rapidly and converge with those of Grevy's zebras (Figure 2).

### 3.3 Microclimatic effects on bias in exposure risk

#### 3.3.1 Frequency of shade in feeding and defecation sites

Grevy's zebras spent a greater proportion of their grazing time in shade than did plains zebras (W = 245, *p* < .05, *N* = 16 Grevy's and 45 plains zebra scan samples). The proportion of grazing time each species spent in shade (37% for Grevy's and 14% for plains) differed from the proportion of dung found in shade (only 5%, or 7 out of 128 dung piles), a mismatch indicating that defecation and feeding behavior cannot both be randomly distributed behaviors for either species.

#### 3.3.2 Embryonation experiments

The surface temperature of dung was much higher in the sun than in the shade (W = 3939, *p* < .001, *N*_Shade_ = 235, *N*_Sun_ = 231; sample size represents the number of measurements taken in each treatment), and both shade and moistening reduced temperature significantly (GLS shade estimate = −23.9, SE = 0.92, t = −26.0, *p* < .001, moistening estimate = −2.9, SE = 0.70, t = −4.1, *p* < .001).

**FIGURE 3:**
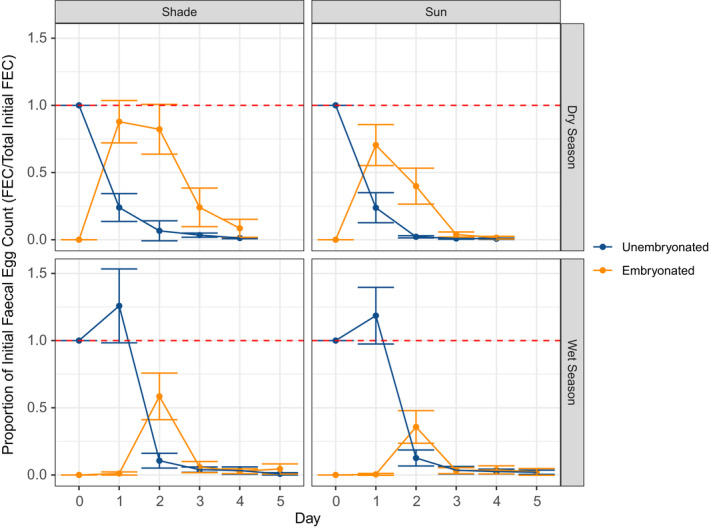
Proportion of initial egg count that embryonated (orange) or remained unembryonated (blue) in the shade vs. sun treatments for the wet and dry season embryonation experiments

We found a strong effect of shade on embryonation. The area under the curve for proportion of initial egg count embryonated over time was greater for all samples in a shade treatment than in a sun treatment (W = 325, *p* < .05, *N*_Shade_ = 40, *N*_Sun_ = 40; Figure S3). The area under the curve was also higher in all samples in the dry season than in the wet season (W = 915, *p* < .001, *N*_Dry_ = 24, *N*_Wet_ = 80). We found that the number of unembryonated eggs plummeted on day 1 (dry season) or day 2 (wet season), concurrently with an increase in embryonated eggs, suggesting that most eggs either died (and disintegrated enough that they were not detectable by salt flotation) or embryonated within the first couple of days of deposition (Figure 3). The effect of shade on the proportion of eggs embryonating was higher in the dry season (Figure 3), most likely because of the lack of humidity (W = 32791, *p* < .001, *N* = 154 humidity measurements in the January dry season, 266 in the July wet season; mean relative humidity was 60.63% ± 1.25 in July, and 37.21% ± 1.51 in January). However, the proportion of the initial egg count that embryonated was higher in January than in July, regardless of treatment, in part due to earlier embryonation onset (Figure 3). While mean temperature did not differ between experimental seasons (19.66°C in January vs. 19.38°C in July; W = 19563, *p* = .44, sample sizes the same as for humidity measurements), daily temperature fluctuations were more variable in the dry season (Figure S2), a condition associated with earlier embryonation in other nematodes (Saunders et al., 2002).

## 4 DISCUSSION

We found evidence for interspecific differences in exposure risk to fecal‐orally transmitted parasites between zebras with different degrees of water dependence. Home ranges of plains zebra groups were closer on average to water points than those of Grevy's zebras, except for male Grevy's zebras, which compete to establish territories close to water. Dung density decreased with distance from water and was higher in areas plains zebras used for grazing than in random areas, whereas Grevy's zebras grazed in areas with dung density on par with that generally found on the landscape. Our results indicate that plains zebras experience a higher risk of exposure to gastrointestinal nematodes than do Grevy's zebras, particularly female and juvenile Grevy's zebras. While we assumed that dung density is highly correlated with the density of infective larvae on the landscape, this should be confirmed in future studies using counts of infective larvae in grass clippings. Further, our dataset included no lactating Grevy's zebra females with young foals, which have been shown to drink as frequently as plains zebras and to restrict their ranging closer to water than other Grevy's zebras (Ginsberg, 1988; Rubenstein, 1986, 1994). Lactating Grevy's zebras are therefore likely to have increased exposure risk and future studies incorporating variation introduced by female reproductive status would add useful nuance to our understanding of host–parasite dynamics in wild equids.

The key role that proximity to water plays in host–parasite dynamics in this system was further supported by our comparison of parasitism at the intraspecific level; FECs declined the farther an individual's home range lay from water, irrespective of species. Surface water serves as an attraction point for many species, and its effects on concentrating animals, their feces, and/or intermediate hosts and vectors, promoting parasite transmission, have been seen in many studies, including on red deer (Vicente et al., 2006), big‐horn sheep (Whiting et al., 2009), and the willow tit and crested tit (Krama et al., 2015). Such effects were recently demonstrated for a suite of herbivore species in our study area, with a particularly strong increase in parasite exposure risk close to water for elephants and cattle (Titcomb et al., 2021). Zebra dung density was not shown to change with distance from water, but this was based on 150‐m long transects; our study builds on this to show that, when measured at a greater spatial scale, zebra dung density declines with distance from water up to about a kilometer away. Similar dynamics may be produced by other attraction points on the landscape, many of which may also draw in multiple species, such as carcasses (Gonzálvez et al., 2021), flower patches visited by various pollinators (Graystock et al., 2015), or stopover sites for migratory birds (Emmenegger et al., 2018). However, using this principle to understand transmission dynamics in a multi‐host, multi‐parasite system requires considerable knowledge of the study system and host–parasite dynamics for each member of the transmission network (Streicker et al., 2013).

The infectivity of dung, and not just its density, is also an important influence on exposure risk. Our embryonation experiments showed that nematodes embryonate with higher probability in the shade than in the sun. However, only a very low proportion of zebra dung on the landscape (5%) was in shade, making the microclimatic effects of shade unlikely to be highly influential on exposure risk dynamics. Our field embryonation experiments contributed to gaps in our understanding of the microclimatic conditions that favor strongyle nematode development. Previous experiments found that adding stochasticity to smooth daily temperature cycles led to earlier embryonation onset in nematodes (Saunders et al., 2002), and our results further indicated that greater *extremity* in temperature fluctuations may hasten embryonation. Daily temperature fluctuations in our dry season field experiments were higher than in the wet season (SI2) and were more than twice the amplitude trialed by Saunders et al. (2002) in the laboratory. Some nematodes (e.g., *Nematodirus battus*) require cooling before an increase in temperature to embryonate (van Dijk & Morgan, 2010). If extreme fluctuations in temperature reduce the survival probabilities of the unembryonated egg, a rapid embryonation and development strategy to reach the infective stage sooner may pay off. Further, previous studies have not tested whether equine strongyle eggs are resistant or susceptible to desiccation (Nielsen et al., 2007). Moistening dung had a negligible effect on embryonation rates in our experiments, suggesting resistance to desiccation, but we did not measure moisture levels inside the dung balls. Equine strongyle eggs have been reported to be heat‐resistant but have only been tested in the laboratory at temperatures of up to 38°C (Nielsen et al., 2007). Our dung piles in the sun treatments reached surface temperatures well above this temperature. The strong effect of sun on embryonation and the great difference in temperature between dung in the sun and shade suggests that in tropical areas, strongyle eggs in the field may effectively be heat‐sensitive.

Consistent discrepancies in exposure risk between related hosts may lead to diverging evolutionary trajectories in the host–parasite arms race as optimal immune strategies vary with the rate of infection. For example, Grevy's zebras appear to produce more immunoglobulin A (IgA), a molecule that may act to provide greater resistance and/or tolerance (in the form of tissue repair) to gastrointestinal nematodes, than do plains zebras (Tombak et al., 2020). This suggests an interspecific divergence in immune investment strategy and may in fact be an alternative explanation (or an additional explanation, along with lower exposure risk) for the lower FECs reported in Grevy's zebras (Rubenstein, 2010). In addition, the present study found that the FEC response to distance from water was shallower and more linear for Grevy's zebras than for plains zebras, suggesting a potentially reduced sensitivity to exposure risk in the Grevy's zebra. A lower vulnerability to GINs would be a hopeful state of affairs for the endangered Grevy's zebra, whose distribution in the past few decades has shifted into much greater sympatry with the plains zebra (Moehlman, 2002; Rubenstein et al., 2016), and therefore into areas with greater zebra dung density. The establishment of artificial water holes has been linked to population crashes of arid‐adapted ungulates, such as the sable antelope in South Africa's Kruger National Park, in response to an influx of mesic‐adapted herbivores and predators (Harrington et al., 1999). Our findings suggest an increase in parasite exposure risk may accompany these challenges and adds support to management practices that maintain habitat heterogeneity and arid habitat refugia in the interest of preserving biodiversity.

Our study demonstrates that host density and exposure risk to parasites do not always have a straightforward relationship. Neither defecation nor feeding and ranging behavior were randomly distributed on the landscape, but were associated with proximity to water and suggested interspecific differences in exposure risk. The findings highlight water dependence as an important consideration for comparisons of parasite pressure on related hosts with divergent requirements for surface water, such as sable antelope and East African oryx, Grant's and Thomson's gazelles, or the greater kudu and common eland (Kihwele et al., 2020). In many cases, the extent to which parasite species are shared between sympatric host species is unknown. Even then, an understanding of host behaviors that shape exposure risk may help to predict the vulnerabilities of each host (Altizer et al., 2003; Barron et al., 2015).

## CONFLICT OF INTEREST

None of the authors have conflicts of interest to declare.

## AUTHOR CONTRIBUTIONS

**Kaia J. Tombak:** Conceptualization (equal); Data curation (lead); Formal analysis (lead); Funding acquisition (supporting); Investigation (equal); Methodology (equal); Project administration (equal); Visualization (lead); Writing – original draft (lead); Writing – review & editing (lead). **Laurel A. Easterling:** Conceptualization (supporting); Investigation (equal); Writing – review & editing (supporting). **Lindsay Martinez:** Investigation (equal); Writing – review & editing (supporting). **Monica S. Seng:** Investigation (equal); Writing – review & editing (supporting). **Liana F. Wait:** Formal analysis (supporting); Investigation (equal); Writing – review & editing (supporting). **Daniel I. Rubenstein:** Conceptualization (equal); Formal analysis (supporting); Funding acquisition (lead); Methodology (equal); Project administration (equal); Supervision (lead); Writing – original draft (supporting); Writing – review & editing (supporting).

## Supporting information

Fig S1‐S3

## Data Availability

All data used for the analyses in this manuscript are available on Dryad at: https://doi.org/10.5061/dryad.xd2547djk.

## References

[ece38693-bib-0001] Altizer, S. , Nunn, C. L. , Thrall, P. H. , Gittleman, J. L. , Antonovics, J. , Cunningham, A. A. , Dobson, A. P. , Ezenwa, V. , Jones, K. E. , Pedersen, A. B. , Poss, M. , & Pulliam, J. R. C. (2003). Social organization and parasite risk in mammals: Integrating theory and empirical studies. Annual Review of Ecology Evolution and Systematics, 34, 517–547. 10.1146/annurev.ecolsys.34.030102.151725

[ece38693-bib-0002] Altmann, J. (1973). Observational study of behavior: Sampling methods. Behaviour, 49(3), 227–267. 10.1163/156853974x00534 4597405

[ece38693-bib-0003] Arneberg, P. , Skorping, A. , Grenfell, B. , & Read, A. F. (1998). Host densities as determinants of abundance in parasite communities. Proceedings of the Royal Society of London B: Biological Sciences, 265, 1283–1289. 10.1098/rspb.1998.0431

[ece38693-bib-0004] Barron, D. G. , Gervasi, S. S. , Pruitt, J. N. , & Martin, L. B. (2015). Behavioral competence: How host behaviors can interact to influence parasite transmission risk. Current Opinion in Behavioral Sciences, 6, 35–40. 10.1016/j.cobeha.2015.08.002

[ece38693-bib-0005] Becker, D. C. , & Ginsberg, J. R. (1990). Mother‐infant behaviour of wild Grevy’s zebra: Adaptations for survival in semi‐desert East Africa. Animal Behaviour, 40, 1111–1118. 10.1016/S0003-3472(05)80177-0

[ece38693-bib-0006] Bowman, D. D. , Lynn, R. C. , & Eberhard, M. L. (2003). Georgis’ parasitology for veterinarians (8th ed., pp. 422). Saunders.

[ece38693-bib-0007] Brashares, J. S. , & Arcese, P. (1999). Scent marking in a territorial African antelope: II. The economics of marking with faeces. Animal Behaviour, 57(1), 11–17. 10.1006/anbe.1998.0942 10053067

[ece38693-bib-0008] Brooks, M. E. , Kristensen, K. , Benthem, K. J. , Magnusson, A. , Berg, C. W. , Nielsen, A. , Skaug, H. J. , Mächler, M. , & Bolker, B. M. (2017). glmmTMB balances speed and flexibility among packages for zero‐inflated generalized linear mixed modeling. The R Journal, 2(9), 378–400. 10.32614/RJ-2017-066

[ece38693-bib-0009] Caylor, K. K. , Gitonga, J. , & Martins, D. J. (2017). Mpala Research Centre meteorological and hydrological dataset. Mpala Research.

[ece38693-bib-0010] Caylor, K. K. , Gitonga, J. , & Martins, D. J. (2018). Mpala Research Centre meteorological and hydrological dataset. Mpala Research.

[ece38693-bib-0011] Crall, J. P. , Stewart, C. V. , Berger‐Wolf, T. Y. , Rubenstein, D. I. , & Sundaresan, S. R. (2013). HotSpotter: Patterned species instance recognition. 2013 IEEE Workshop on Applications of Computer Vision (WACV), pp. 230–237. 10.1109/WACV.2013.6475023

[ece38693-bib-0012] Dobson, A. P. (1990). Models for multi‐species parasite‐host communities. In G. W. Esch , A. O. Bush , & J. M. Aho (Eds.), Parasite communities: Patterns and processes (pp. 261–288). Springer.

[ece38693-bib-0013] Emmenegger, T. , Bauer, S. , Hahn, S. , Müller, S. B. , Spina, F. , & Jenni, L. (2018). Blood parasites prevalence of migrating passerines increases over the spring passage period. Journal of Zoology, 306(1), 23–27. 10.1111/jzo.12565

[ece38693-bib-0014] Ezenwa, V. O. (2003). Habitat overlap and gastrointestinal parasitism in sympatric African bovids. Parasitology, 126(Pt 4), 379–388. 10.1017/S0031182002002913 12741517

[ece38693-bib-0015] Ezenwa, V. O. (2004a). Host social behavior and parasitic infection: A multifactorial approach. Behavioral Ecology, 15(3), 446–454. 10.1093/beheco/arh028

[ece38693-bib-0016] Ezenwa, V. O. (2004b). Selective defecation and selective foraging: Antiparasite behavior in wild ungulates? Ethology, 110(11), 851–862. 10.1111/j.1439-0310.2004.01013.x

[ece38693-bib-0017] Fischhoff, I. R. , Sundaresan, S. R. , Cordingley, J. , & Rubenstein, D. I. (2007). Habitat use and movements of plains zebra (*Equus burchelli*) in response to predation danger from lions. Behavioral Ecology, 18(4), 725–729. 10.1093/beheco/arm036

[ece38693-bib-0018] Fleurance, G. , Duncan, P. , Fritz, H. , Cabaret, J. , Gordon, J. , & Importance, I. J. (2005). Importance of nutritional and anti‐parasite strategies in the foraging decisions of horses: An experimental test. Oikos, 110, 602–612. 10.1111/j.0030-1299.2005.13428.x

[ece38693-bib-0019] Foreyt, J. W. (2001). Veterinary parasitology: reference manual (5th ed.). Iowa State University Press.

[ece38693-bib-0020] Ginsberg, J. R. (1988). Social organization and mating strategies of an arid adapted equid: The Grevy’s Zebra. Princeton University.

[ece38693-bib-0021] Ginsberg, J. R. (1989). The ecology of female behaviour and male reproductive success in the Grevy’s zebra, *Equus grevyi* . Symposia of the Zoological Society of London, 61, 89–110.

[ece38693-bib-0022] Gonzálvez, M. , Martínez‐Carrasco, C. , & Moleón, M. (2021). Understanding potential implications for non‐trophic parasite transmission based on vertebrate behavior at mesocarnivore carcass sites. Veterinary Research Communications, 45(4), 261–275. 10.1007/s11259-021-09806-2 34176034 PMC8235911

[ece38693-bib-0023] Graystock, P. , Goulson, D. , & Hughes, W. O. H. (2015). Parasites in bloom: Flowers aid dispersal and transmission of pollinator parasites within and between bee species. Proceedings of the Royal Society B: Biological Sciences, 282(1813), 20151371. 10.1098/rspb.2015.1371 PMC4632632 26246556

[ece38693-bib-0024] Grenfell, B. T. , Smith, G. , & Anderson, R. M. (1987). A mathematical model of the population biology of *Ostertagia ostertagi* in calves and yearlings. Parasitology, 95(2), 389–406. 10.1017/S0031182000057826 3696772

[ece38693-bib-0025] Harrington, R. , Owen‐Smith, N. , Viljoen, P. C. , Biggs, H. C. , Mason, D. R. , & Funston, P. (1999). Establishing the causes of the roan antelope decline in the Kruger National Park. South Africa. Biological Conservation, 90(1), 69–78. 10.1016/S0006-3207(98)00120-7

[ece38693-bib-0026] Hartig, F. (2018). DHARMa: Residual diagnostics for hierarchical (multi‐level/mixed) regression models. R Package Version 0.2.0. https://cran.r‐project.org/package=DHARMa

[ece38693-bib-0027] Herd, R. P. (1992). Performing equine fecal egg counts. Veterinary Medicine, 87, 240–244.

[ece38693-bib-0028] Kihwele, E. S. , Mchomvu, V. , Owen‐Smith, N. , Hetem, R. S. , Hutchinson, M. C. , Potter, A. B. , Olff, H. , & Veldhuis, M. P. (2020). Quantifying water requirements of African ungulates through a combination of functional traits. Ecological Monographs, 90(2), 1–11. 10.1002/ecm.1404

[ece38693-bib-0029] Klingel, H. (1972). Social behaviour of African Equidae. Zoologica Africana, 7(1), 175–185. 10.1080/00445096.1972.11447438

[ece38693-bib-0030] Krama, T. , Krams, R. , Cīrule, D. , Moore, F. R. , Rantala, M. J. , & Krams, I. A. (2015). Intensity of haemosporidian infection of parids positively correlates with proximity to water bodies, but negatively with host survival. Journal of Ornithology, 156(4), 1075–1084. 10.1007/s10336-015-1206-5

[ece38693-bib-0031] Krecek, R. C. , Reinecke, R. K. , & Malan, F. S. (1987). Studies on the parasites of zebras. V. Nematodes of the Burchell’s and Hartmann’s mountain zebras from the Etosha National Park, South West Africa/Namibia. Onderstepoort Journal of Veterinary Research, 54(1), 71–78. 3587931

[ece38693-bib-0032] Lamoot, I. , Callebaut, J. , Degezelle, T. , Demeulenaere, E. , Laquière, J. , Vandenberghe, C. , & Hoffmann, M. (2004). Eliminative behaviour of free‐ranging horses: Do they show latrine behaviour or do they defecate where they graze? Applied Animal Behaviour Science, 86, 105–121. 10.1016/j.applanim.2003.12.008

[ece38693-bib-0033] Matthee, S. , Krecek, R. C. , & Milne, S. A. (2000). Prevalence and biodiversity of helminth parasites in donkeys from South Africa. The Journal of Parasitology, 86, 756–762. 10958452 10.1645/0022-3395(2000)086[0756:PABOHP]2.0.CO;2

[ece38693-bib-0034] Moehlman, P. D. (1985). The odd‐toed ungulates: Order Perissodactyla. In R. E. Brown , & D. W. Macdonald (Eds.), Social odours in mammals (Vol. 2, pp. 531–549). Clarendon Press.

[ece38693-bib-0035] Moehlman, P. D. (2002). Equids: Zebras, asses and horses. International Union for the Conservation of Nature. http://books.google.com/books?hl=en&lr=&id=8Bj4q83ry1QC&pgis=1

[ece38693-bib-0036] Morand, S. , & Poulin, R. (1998). Density, body mass and parasite species richness of terrestrial mammals. Evolutionary Ecology, 12, 717–727.

[ece38693-bib-0037] Nielsen, M. K. , Baptiste, K. E. , Tolliver, S. C. , Collins, S. S. , & Lyons, E. T. (2010). Analysis of multiyear studies in horses in Kentucky to ascertain whether counts of eggs and larvae per gram of feces are reliable indicators of numbers of strongyles and ascarids present. Veterinary Parasitology, 174(1–2), 77–84. 10.1016/j.vetpar.2010.08.007 20850927

[ece38693-bib-0038] Nielsen, M. K. , Kaplan, R. M. , Thamsborg, S. M. , Monrad, J. , & Olsen, S. N. (2007). Climatic influences on development and survival of free‐living stages of equine strongyles: Implications for worm control strategies and managing anthelmintic resistance. Veterinary Journal, 174(1), 23–32. 10.1016/j.tvjl.2006.05.009 16815051

[ece38693-bib-0039] Parham, J. , Crall, J. , Rubenstein, D. I. , Holmberg, J. , Berger‐Wolf, T. , & Stewart, C. (2018). An animal detection pipeline for identification. In IEEE Winter Conference on Applications of Computer Vision (WACV) (pp. 1–9).

[ece38693-bib-0040] Poulin, R. (2010). Decay of similarity with host phylogenetic distance in parasite faunas. Parasitology, 137, 733–741. 10.1017/S0031182009991491 19849890

[ece38693-bib-0041] QGIS Development Team . (2018). QGIS geographic information system. Open Source Geospatial Foundation Project.

[ece38693-bib-0042] R Core Team . (2018). R: A language and environment for statistical computing. R Foundation for Statistical Computing.

[ece38693-bib-0043] Rose, H. , Wang, T. , Dijk, J. V. , & Morgan, E. R. (2015). GLOWORM‐FL: A simulation model of the effects of climate and climate change on the free‐living stages of gastro‐intestinal nematode parasites of ruminants. Ecological Modelling, 297, 232–245. 10.1016/j.ecolmodel.2014.11.033

[ece38693-bib-0044] Rubenstein, D. I. (1986). Ecology and sociality in horses and zebras. In D. I. Rubenstein , & R. W. Wrangham (Eds.), Ecological aspects of social evolution (pp. 282–302). Princeton University Press.

[ece38693-bib-0045] Rubenstein, D. I. (1994). The ecology of female social behaviour in horses, zebras and asses. In P. Jarman , & A. Rossiter (Eds.), Animal societies: Individuals, interaction and organisation (pp. 13–28). Kyoto University Press.

[ece38693-bib-0046] Rubenstein, D. I. (2010). Ecology, social behavior, and conservation in zebras. In R. Macedo (Ed.), Advances in the study of behavior: Behavioral ecology of tropical animals (1st ed., Vol. 42, pp. 231–258). Elsevier Inc. 10.1016/S0065-3454(10)42007-0

[ece38693-bib-0047] Rubenstein, D. I. , Low Mackey, B. , Davidson, Z. , Kebede, F. , & King, S. R. (2016). Equus grevyi. The IUCN Red List of Threatened Species 2016, e.T7950A89. 10.2305/IUCN.UK.2016-3.RLTS.T7950A89624491.en

[ece38693-bib-0048] Saunders, L. M. , Tompkins, D. M. , & Hudson, P. J. (2002). Stochasticity accelerates nematode egg development. The Journal of Parasitology, 88(6), 1271. 10.2307/3285511 12537129

[ece38693-bib-0049] Scialdo, R. C. , Reinecke, R. K. , & de Vos, V. (1982). Seasonal incidence of helminths in the Burchell’s zebra. Onderstepoort Journal of Veterinary Research, 49, 127–130. 7177583

[ece38693-bib-0050] Scialdo‐Krecek, R. C. , Reinecke, R. K. , & Malan, F. S. (1983). Studies on the parasites of zebras. III. Nematodes of the mountain zebra from the farm “Kelpie” and the Namib‐Naukluft Park, South West Africa/Namibia. Onderstepoort Journal of Veterinary Research, 50, 283–290. 6676691

[ece38693-bib-0051] Signorell et mult. al., A. (2019). DescTools: Tools for descriptive statistics.

[ece38693-bib-0052] Silveira, S. (2019). Dung avoidance in zebra: The trade‐off between nutritional advantages and parasitic risks. Princeton University.

[ece38693-bib-0053] Streicker, D. G. , Fenton, A. , & Pedersen, A. B. (2013). Differential sources of host species heterogeneity influence the transmission and control of multihost parasites. Ecology Letters, 16(8), 975–984. 10.1111/ele.12122 23714379 PMC4282463

[ece38693-bib-0054] Titcomb, G. , Mantas, J. N. , Hulke, J. , Rodriguez, I. , Branch, D. , & Young, H. (2021). Water sources aggregate parasites with increasing effects in more arid conditions. Nature Communications, 12, 1–12. 10.1038/s41467-021-27352-y PMC8642388 34862389

[ece38693-bib-0055] Titcomb, G. , Pansu, J. , Hutchinson, M. C. , Tombak, K. J. , & Young, H. S. (2020). Metabarcoding reveals parasite communities and their overlaps in large mammalian herbivores. Ecological Society of America.

[ece38693-bib-0056] Tombak, K. J. , Budischak, S. A. , Hauck, S. , Martinez, L. A. , & Rubenstein, D. I. (2020). The non‐invasive measurement of faecal immunoglobulin in African equids. International Journal for Parasitology: Parasites and Wildlife, 12, 105–112. 10.1016/j.ijppaw.2020.05.005 32528845 PMC7283094

[ece38693-bib-0057] Tombak, K. J. , Hansen, C. B. , Kinsella, J. M. , Pansu, J. , Pringle, R. M. , & Rubenstein, D. I. (2021). The gastrointestinal nematodes of plains and Grevy’s zebras: Phylogenetic relationships and host specificity. International Journal for Parasitology: Parasites and Wildlife, 16, 228–235. 10.1016/j.ijppaw.2021.10.007 34712556 PMC8529100

[ece38693-bib-0058] van Dijk, J. , & Morgan, E. R. (2010). Variation in the hatching behaviour of *Nematodirus battus*: Polymorphic bet hedging? International Journal for Parasitology, 40, 675–681. 10.1016/j.ijpara.2009.11.002 19944106

[ece38693-bib-0059] VanderWaal, K. , Omondi, G. P. , & Obanda, V. (2014). Mixed‐host aggregations and helminth parasite sharing in an East African wildlife‐livestock system. Veterinary Parasitology, 205(1–2), 224–232. 10.1016/j.vetpar.2014.07.015 25086496

[ece38693-bib-0060] Vicente, J. , Fernández De Mera, I. G. , & Gortazar, C. (2006). Epidemiology and risk factors analysis of elaphostrongylosis in red deer (*Cervus elaphus*) from Spain. Parasitology Research, 98(2), 77–85. 10.1007/s00436-005-0001-2 16265599

[ece38693-bib-0061] Wambwa, E. N. , Ogara, W. O. , & Mudakha, D. (2004). A comparative study of gastrointestinal parasites between ranched and free ranging Burchell’s zebra (*Equus burchelli antiquorum*) in Isiolo district, Kenya. Journal of Veterinary Science, 5(3), 215–220. 10.4142/jvs.2004.5.3.215 15365235

[ece38693-bib-0062] Whiting, J. C. , Bowyer, R. T. , & Flinders, J. T. (2009). Annual use of water sources by reintroduced Rocky Mountain bighorn sheep *Ovis canadensis canadensis*: Effects of season and drought. Acta Theriologica, 54(2), 127–136. 10.1007/BF03193168

[ece38693-bib-0063] Young, T. P. , Okello, B. , Kinyua, D. , & Palmer, T. (1997). KLEE: A long‐term multi‐species herbivore exclusion experiment in Laikipia, Kenya. African Journal of Range and Forage Science, 14, 92–104. 10.1080/10220119.1997.9647929

