# Supplementary material for: Divergent water requirements partition exposure risk to parasites in wild equids
Source: Ecol Evol. 2022 Mar 14;12(3):e8693. doi: 10.1002/ece3.8693 (PMC8928873; doi:10.1002/ece3.8693)

Supplementary Information

Figure S1: Home ranges, sightings, and centroids mapped in southern Mpala for plains (left) and Grevy's zebras (right) in June-July 2016 and June-July 2018.

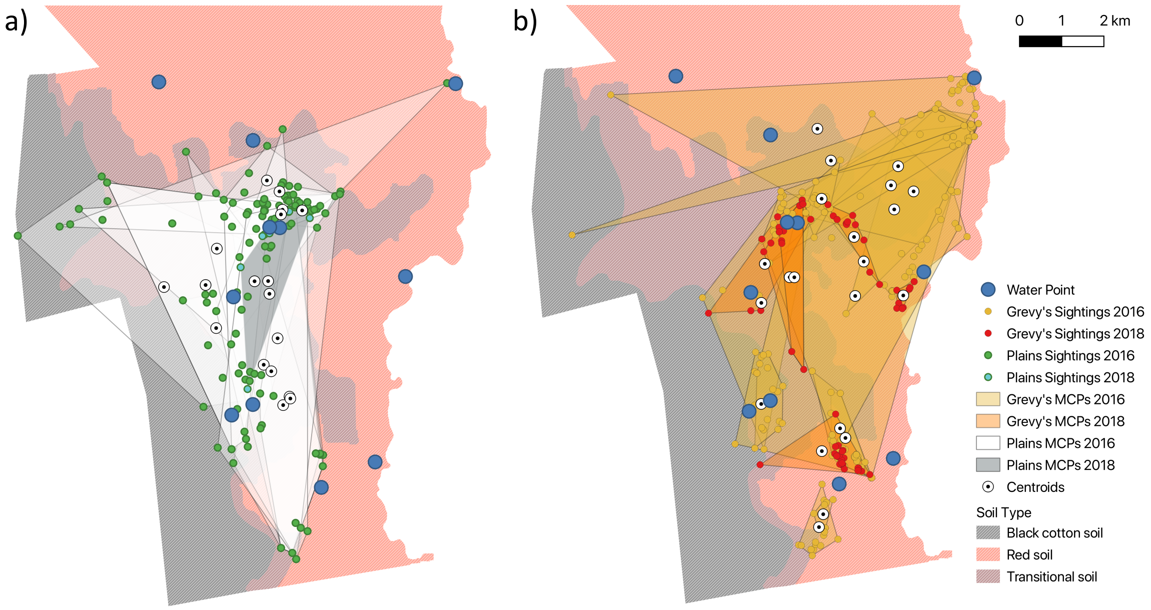

Soil map from Mpala Research Centre

Figure S2: Daily fluctuations in temperature were more variable and extreme in January than in July. Note that hourly temperature data were available at Mpala Research Centre for the entire span of the July 2016 experiments but only for the first six days of the January 2017 experiments.

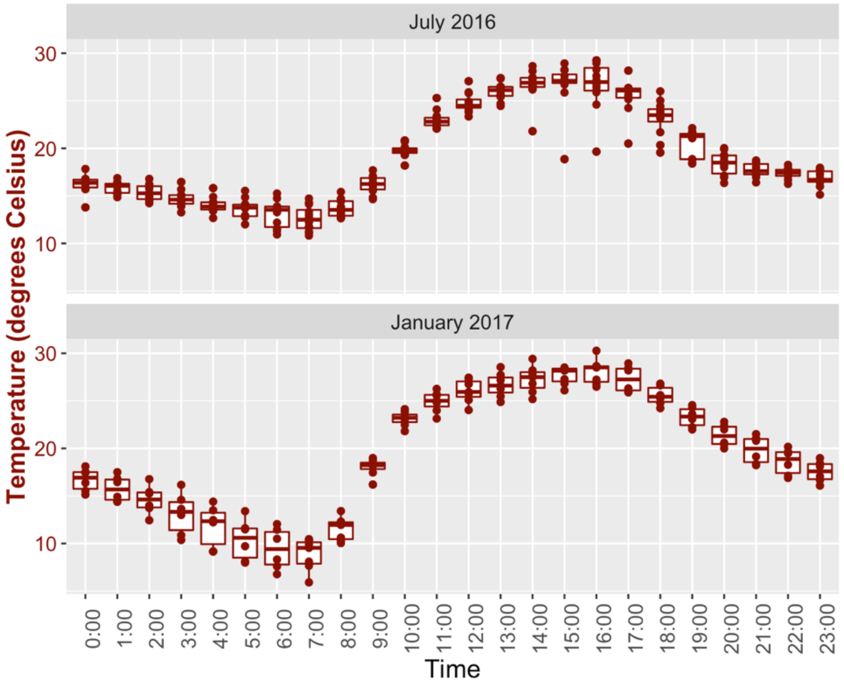

Figure S3: Full results of each treatment from each season of embryonation experiments. Note that because of a slight difference in design, where the wet season experiments were modelling increasingly worse conditions whereas the dry season experiments followed a block design (Fig. 1), the sun-bare ground treatment was only conducted in the wet season and the sun-wet treatment only in the dry season. In each season, the main effects on embryonation came from sun vs. shade and from season. However, moistening the dung boosted embryonation probability in the sun on day 2 in the dry season experiments.

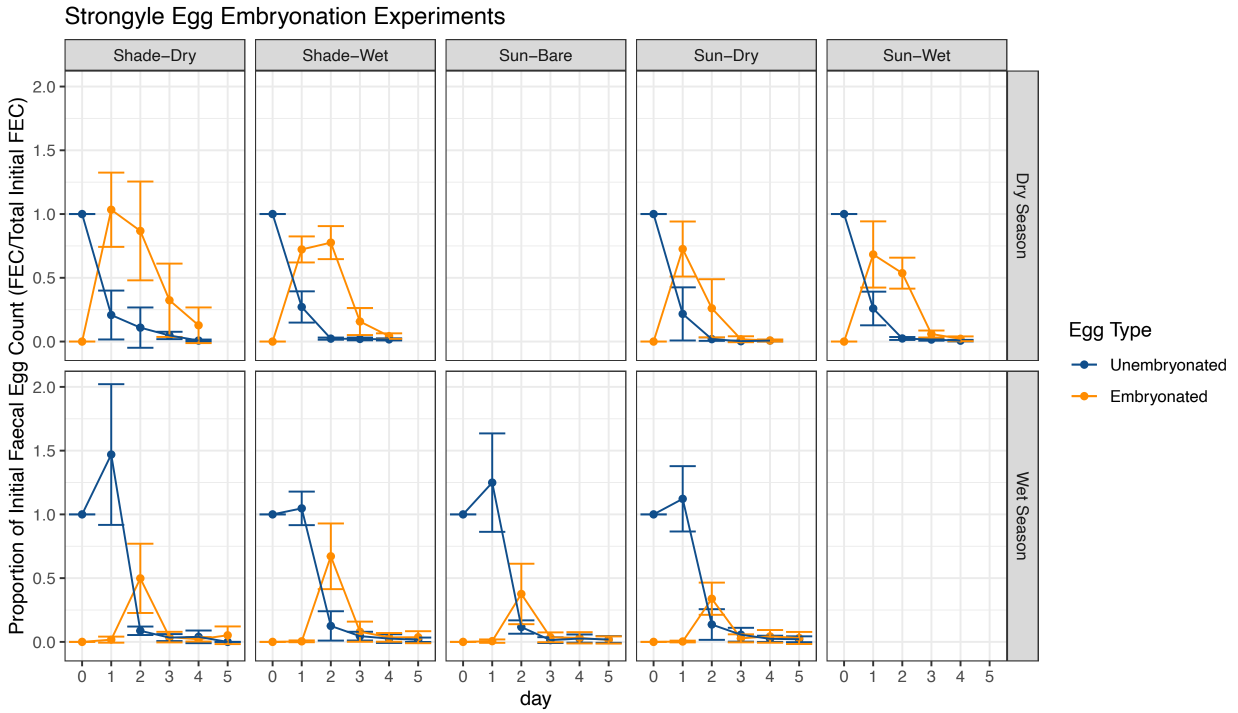

Supplement: Supplementary file 1 — Fig S1‐S3 [file ECE3-12-e8693-s001.pdf]
